# Effects of treatments for drooling on caries risk in children and adolescents with cerebral palsy

**DOI:** 10.4317/medoral.22729

**Published:** 2019-03

**Authors:** Gabriela-Mancia Gutierrez, Vanessa-Lira Siqueira, Juan-Pablo Loyola-Rodriguez, Michele-Baffi Diniz, Renata-Oliveira Guaré, Ana-Cristina-Fernandes-Maria Ferreira, Maria-Teresa-Botti-Rodrigues Santos

**Affiliations:** 1 Ph.D student, Postgraduate Program in Dentistry, Cruzeiro do Sul University, Rua Galvão Bueno, 868 - Liberdade, São Paulo - SP, Brazil; 2 MSc student, Postgraduate Program in Dentistry, Cruzeiro do Sul University, Rua Galvão Bueno, 868 - Liberdade, São Paulo - SP, Brazil; 3 Escuela Superior de Odontología, Universidad Autónoma de Guerrero, Morelos s/n, Granjas del Marqués, 39890 Acapulco, Gro., México; 4 Assistant Professor, Pediatric Dentistry, Postgraduate Program in Dentistry, Cruzeiro do Sul University, Rua Galvão Bueno, 868 - Liberdade, São Paulo - SP, Brazil; 5 Associate Professor, Pediatric Dentistry, Postgraduate Program in Dentistry, Cruzeiro do Sul University, Rua Galvão Bueno, 868 - Liberdade, São Paulo - SP, Brazil; 6 Ph.D student, Postgraduate Program in Dentistry, Cruzeiro do Sul University, Rua Galvão Bueno, 868 - Liberdade, São Paulo - SP, Brazil; 7 Associate Professor, Individuals with Special Needs, Postgraduate Program in Dentistry, Cruzeiro do Sul University. Rua Galvão Bueno, 868 - Liberdade, São Paulo - SP, Brazil

## Abstract

**Background:**

Neuromuscular impairment makes individuals with cerebral palsy (CP) more prone to drooling. Among the treatment options, there are procedures that interfere with saliva production. It is imperative to evaluate the effect of the different modalities since the reduction in salivary flow rate/production may exacerbate the risk of dental caries.

**Material and Methods:**

The aim of this study was to compare the effects of different treatments for drooling on caries risk and salivary parameters in children and adolescents with CP.

**Study design:**

A total of 142 children and adolescents with CP, aged 6 to 18 years, were assigned to groups based on the different treatments they had received for drooling: G1—anticholinergic drugs (n = 18), G2—botulinum toxin injection (n = 16), G3—salivary glands surgery (n = 16), G4—no treatment (n = 42), and G5—non-drooling subjects (n = 50). All participants were evaluated on the Simplified Oral Hygiene Index, and for the prevalence of dental caries (decayed, missing, and filled teeth index and white spot lesions). Unstimulated whole saliva was collected, and salivary flow rate and osmolality were measured. Chi-square, ANOVA and Poisson regression were calculated. Prevalence ratios and their respective 95 % confidence intervals were obtained. The significance level was fixed at 5%.

**Results:**

No differences were found in the decayed, missing, and filled teeth index (*p* = 0.128) and Simplified Oral Hygiene Index (*p* = 0.674) among the different groups. G3 presented significantly higher percentages of WSL (*p*<0.001), lower values of salivary flow rate (*p*<0.001), and higher values of osmolality (*p*<0.001). The white spot lesion prevalence ratio was higher only for G3 (Prevalence ratio = 14.36; IC 95% = 4.64-44.40; *p*<0.001).

**Conclusions:**

Children and adolescents with CP who had received surgical treatment for drooling exhibited higher number of white spot lesions because of the reduced salivary flow rate and higher salivary osmolality.

** Key words:**Cerebral palsy, saliva, sialorrhea, dental caries, osmolar concentration.

## Introduction

Cerebral palsy (CP) describes a prevalent, clinically important, and identifiable medical condition with non-progressive permanent neuromotor disorders, caused by damage to the immature or developing brain, with consequent activity limitations regarding movement and posture (1). In CP, motor function impairments are frequently accompanied by disturbances of cognition and behavior, and in later life, by the development of musculoskeletal problems, including impairment of oral motor and speech functions (1). Neuromuscular impairment makes individuals with CP prone to drooling (2). 

Saliva, responsible for mechanical cleaning and protective functions, is essential to the maintenance of oral health. Salivary flow rate (SFR) and its buffering action play a critical role in maintaining the pH of saliva (3). Low SFR values and higher osmolality (SO) values are correlated with an increased prevalence of dental caries in individuals with CP (4). Drooling, or sialorrhea, is the involuntary loss of saliva and its components from the mouth; it is considered a pathological condition if persistent after the age of 4 years (5). For individuals with CP, major predisposing factors for drooling include absence of cervical motor control, alterations in swallowing and speech (dysarthria/dyspraxia), dysphagia, anterior open bite, nasal obstruction, and intraoral sensitivity disorder (6,7,8). The prevalence of drooling in CP varies from 10% (6), 40% (5), 48.7% (7), 58% (8) to 60.3% (9), and negatively affects the social, psychological, and physical health of these patients (10).

Numerous treatment modalities for drooling have been proposed by medical teams. The first option presented is usually oral motor therapy to improve lip closure, swallowing, tongue control, cervical motor control, and oral sensation (11). This therapeutic approach is unique in that it treats the etiology of drooling (12). The second approach, the use of anticholinergic drugs, aims to reduce salivary volume without affecting swallowing function. In this context, muscarinic cholinergic receptor blockers are used, such as scopolamine and atropine (13). The third approach is application of botulinum toxin to the parotid and/or submandibular salivary glands, under ultrasound guidance (14). The transient effect of reduction of saliva secretion and drooling is due to inhibition of the discharge of acetylcholine from cholinergic nerve terminals (14). A fourth approach, the surgical treatment modality, is elected when drooling persists after at least six months of conservative therapy in patients with cognitive deficits and children over 6 years of age, when maturation of oral motor function is expected to occur. The best results occur when bilateral submandibular gland excision is performed with parotid duct ligation, reducing pneumonia aspiration (15).

Although the literature regarding drooling in individuals with CP and its therapies is extensive, only one study described the dental health of children with cerebral palsy following sialodochoplasty (16) and another, the use of intraglandular onabotulinum toxin A injection in these patients (17). Moreover, the development of white spot lesions (WSL) in individuals with CP, who have undergone different treatments for drooling, has not been investigated.

Thus, the purpose of this study was to compare the effects of pharmacological therapy, botulinum toxin injection, and salivary gland surgery on salivary parameters and caries risk in children and adolescents with CP. It was hypothesized that individuals with CP who underwent bilateral removal of the submandibular glands with parotid duct ligation would present higher risk for dental caries (WSL) due to the reduction in SFR and increase in SO.

## Material and Methods

-Ethical statement

This study was reviewed and approved by the local Human Research Ethics Committee (Plataforma Brazil # 1.655.830). Written informed consent was obtained from the guardians of each child or adolescent after they were informed about the study.

-Study design

An epidemiological cross-sectional study was performed with children and adolescents with CP who were referred to a specialized physical rehabilitation center in São Paulo, Brazil.

-Participants

Two hundred and twenty-two children and adolescents with a medical diagnosis of CP composed the convenience sample in this study. Data were collected between February and October 2016.

Inclusion criteria were a medical diagnosis of CP; aged 6–18 years; both genders; presence or absence of drooling; submitted or unsubmitted to different modalities of drooling treatments. Participants using anticholinergic drugs to control drooling were required to have taken the medicine without interruption for at least one month prior to clinical examination. For the botulinum toxin group, the injections in submandibular and parotid glands were required to have been applied within three months prior to the dental clinical evaluation. Participants who underwent salivary gland surgery must have done so in 2015 and followed up with an otolaryngologist at least twice during the last six months, with an evaluation of surgical success. Participants who were G-tube fed, and those who exhibited uncooperative behavior during saliva collection and clinical oral examination were excluded.

According to the inclusion/exclusion criteria, the final sample for this study was composed of 142 subjects with CP, who were assigned to one of five groups depending on the treatment they had received for drooling or not: G1—drooling CP subjects who received anticholinergic drugs (n = 18), G2—drooling CP subjects who received botulinum toxin injection (n = 16), G3—drooling CP subjects who received surgical treatment (n = 16) G4—drooling CP subjects who received no treatment (n = 42; positive control), and G5—non-drooling CP subjects (n = 50; negative control).

The drooling treatment proposed by the medical team of the rehabilitation center obeyed a stepwise approach to the management of drooling, i.e., pharmacotherapy, botulinum toxin, and surgery (18).

All children or adolescents who participated in the study received the same preventive protocol (biofilm control, professional prophylaxis, and fluoride therapy) and dental treatment as needed, before the drooling treatment. Data regarding gender, age, medical diagnosis of CP according to the type of movement disorder (spastic or dyskinetic), and clinical pattern of involvement (tetraparesis, diparesis, or hemiparesis) were collected from their medical records.

-Clinical Evaluation

Oral Hygiene Index (OHI-S)

All evaluations were performed in a dental office under appropriated light-reflector illumination in a dental chair, by a single examiner. Six teeth (four posterior and two anterior) were assessed and scored for each individual according to the Simplified Oral Hygiene Index (OHI-S) (19). For the posterior teeth, the first fully erupted tooth distal to the second premolar or primary molar was examined in each quadrant. For maxillary molars, the buccal surfaces were scored, and for mandibular molars, the lingual surfaces were scored. For anterior teeth, the labial surfaces of the maxillary right and mandibular left central incisors were scored. The OHI-S is a combination of visible plaque and dental calculus.

Dental caries assessment 

A single calibrated examiner (weighted kappa = 0.89) performed the clinical examination with the help of an assistant to record data.

Before dental assessment, professional prophylaxis was performed on each patient. A clinical examination was performed with the aid of a light reflector, air/water spray, dental mirror, and WHO probe, according to World Health Organization criteria (20). Prevalence of dental caries was recorded according to the decayed, missing, and filled teeth index (dmf-t and DMF-T indices for primary and permanent dentition, respectively). In children with mixed dentition, dmf-t and DMF-T were recorded together. No radiographic examination was performed.

The presence of WSL was recorded according to International Caries Detection and Assessment System (ICDAS) criteria (21). The presence of distinct visual change in enamel was recorded as ICDAS code 2 (tooth must be viewed wet).

Saliva collection and assessment 

Unstimulated whole saliva was collected during the same period (between 8:00 and 10:00 a.m.), at least one hour after a meal, using one absorbent cotton roll (Salivette®, Sarstedt, Nümbrecht, Germany) under the tongue, for exactly five minutes, as previously described by our research group (22). Saliva samples were immediately frozen on dry ice, transported to the laboratory, and stored at -80°C. Before SFR and SO measurements were taken, samples were centrifuged at 5000 rpm for 5 minutes at 4°C (Hettich Centrifuge, model Universal 320R, Tuttlingen, Germany). SFR was calculated as ml/mim and SO was measured using a freezing point depression osmometer (Model Vapro Vapor Pressure Osmometer 5600, New Instrument, Washington, DC, USA). Prior to measurement, the osmometer was calibrated by the comparison method using certified standard liquids for the calibration of SO (OPTIMOLE TM 290 and 1000 mmol/kg Osmolality Standard ELITech Group WESCOR).

-Statistical analysis

Analyses of descriptive statistics were performed to calculate demographic data. Inferential analyses were performed using Chi-square and ANOVA tests. Poisson regression analysis was performed to identify factors associated with the presence of WSL. Prevalence ratios (PR) and their respective 95 % confidence intervals were obtained. SPSS Statistical Package for the Social Sciences 19.0 (IBM Brazil, SP, Brazil) was used for all analyses, with a significance level of 5%.

## Results

A total of 142 children and adolescents with CP were enrolled in the study; 63.4% were male and 36.6% were female. Mean (± SD) age was 9.8 (± 2.9). The follow-up period (from the beginning of each treatment modality to the clinical examination) ranged from 4.00 to 5.00 months, with an average and standard deviation (SD) of 4.4 ± 0.9, with no statistical differences between the five groups (*p* = 0.105, ANOVA test).

Descriptive statistics regarding gender, age, and types of movement disorders are shown in  Table 1. Eighteen participants (12.7%) had been treated using anticholinergic drugs (G1), 16 (11.3%) had been treated with botulinum toxin injection (G2), and 16 (11.3%) had received surgical treatment (G3). Drooling subjects who received no treatment (42, 29.6%) were positive controls (G4), and non-drooling subjects (50, 35.1%) were considered to be negative controls (G5). The groups were statistically homogeneous regarding gender (*p* = 0.102) and age (*p* = 0.915). Considering the different types of CP movement disorders, the groups differed significantly (*p* < 0.001), and the negative control group (G5) showed the highest percentage of subjects with spastic diparesis (54.0%).

**Table 1:**
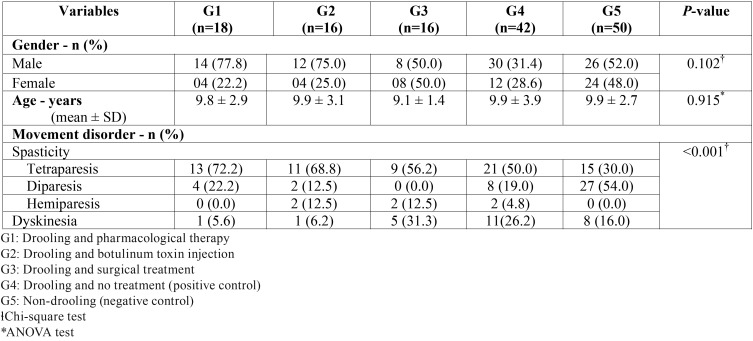
Descriptive characteristics of children and adolescents with CP who were submitted or not to different treatment modalities of drooling.

The power of the study for the sample was calculated as 99.5% and 100.0%, considering the SFR and SO values of the groups, with alpha = 5% (OpenEpi).

 Table 2 shows mean (± SD) for DMF-T index, frequency of WSL, OHI-S, SFR, and SO for all groups. In respect to the DMF-T index (*p* = 0.128) and OHI-S (*p* = 0.674) no significant difference were reported among the groups. Children and adolescents that underwent surgical treatment (G3) presented significantly higher percentages of WSL (*p* < 0.001), lower mean values for SFR (*p* < 0.001), and higher values of SO (*p* < 0.001) than the other treatments modalities and control groups.

**Table 2:**
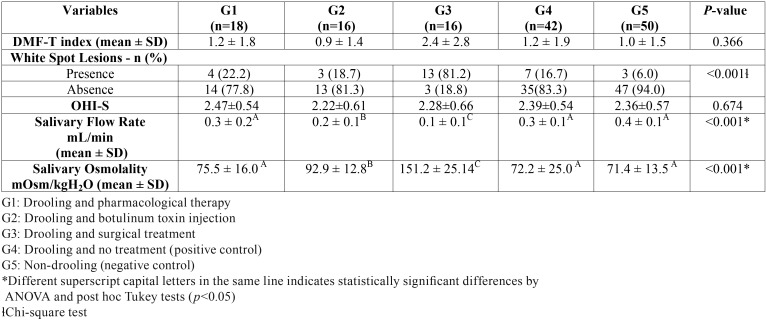
Mean values and standard deviation (±SD) for DMF-T index, salivary flow rate, salivary osmolality and frequency of white spot lesion (WSL) of children and adolescents with CP who were submitted or not to different treatment modalities of drooling.

 Table 3 shows the results of the Poisson regression analysis for the presence of WSL according to characteristics of children and adolescents with CP who did or did not undergo different treatment modalities for drooling. Based on the adjusted model, the prevalence ratio for WSL was observed to be higher for all treatment modalities, and there was statistically significant for the surgical treatment group alone (prevalence ratio = 14.36; IC 95% = 4.64-44.40; *p* < 0.001).

**Table 3:**
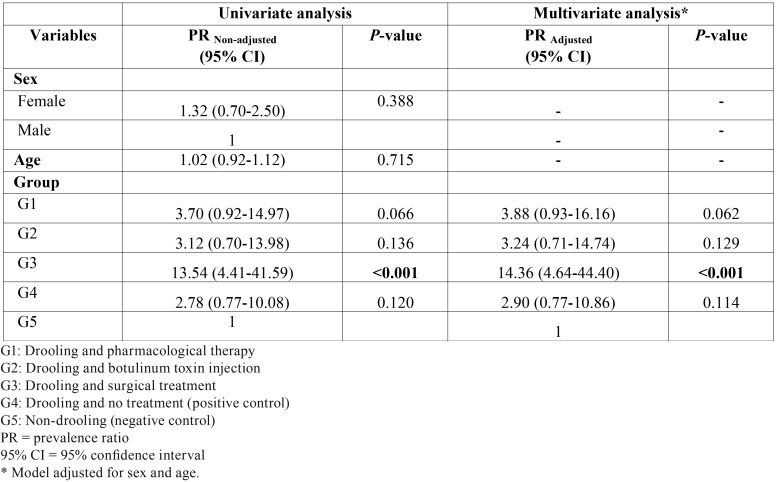
Poisson regression analysis for the presence of white spot lesion (WSL) according to characteristics of children and adolescents with CP who were submitted or not to different treatment modalities of drooling.

## Discussion

The presence of drooling requires treatment, and the literature describes different therapeutic modalities. To our knowledge, this is the first study to evaluate and compare the effects of pharmacological therapy, botulinum toxin injections, and salivary gland surgery in children and adolescents with CP regarding caries risk, considering initial caries lesions (ICDAS code 2) and salivary parameters.

The localization and extent of neuromotor disorder are responsible for the great variability of motor impairment and functional independence of individuals with CP (1). Spastic CP, characterized by increased muscle tone and presence of spasticity, is the most common movement disorder among CP individuals, with a prevalence of 70-80% (1). In the present study, most of the participants (81.7%) presented spasticity; 48.6% of all participants presented with tetraparesis—the highest level of motor impairment—with all limbs compromised, difficulty with cervical motor control, delayed maturation of oral motor function, alterations in swallowing and speech, anterior open bite, nasal obstruction, intraoral sensitivity disorder (2,5) and moderate to severe drooling, as observed in this study. About 28.9% participants presented with diparesis, with upper limb motor control and usually with preserved oral motor functions, which reduced the prevalence of drooling. Consistent with the literature, a higher prevalence of males with CP (63.4%) was observed, but the reasons for which remain unclear (22).

It should be emphasized that the presence of dysphagia and increased saliva production may increase the risk for aspiration pneumonia, and consequently, pulmonary complications, which are the main indications for treatment of drooling in individuals with CP (2). The constant presence of wet clothing, perioral dermatitis, and infections require treatment (10,23). Drooling is also distressing for patients with CP, with negative consequences on their social, emotional, and psychological health. Once treated, patients present improvement in self-esteem and social interactions, and reduction in daily care needs (23). The high prevalence of drooling in individuals with CP motivates clinicians to offer effective and individualized treatment choices to improve health and quality of life (11). However, limitations exist for all treatments for drooling, and except for surgical treatment, they are all temporary. Furthermore, all treatment modalities target the consequences of drooling, reducing salivary volume (24), but fail to address the predisposing factors or causes (12).

Regarding dental caries, significant limitations should be discussed when using the DMF-T index proposed by World Health Organization (20) in oral epidemiological surveys. This index does not provide information related to non-cavitated caries lesions (25). If assessed in their early stages, caries lesions can be arrested through preventive programs, which can also lower the costs related to restorative treatment (26). In the present investigation, the DMF-T index was used in association with the ICDAS criteria (code 2) to evaluate incipient caries lesions in this population.

It should be noticed that the DMF-T index (total number of teeth that are decayed, missing or filled) did not statistically differ among the groups. This fact could be explained by the inclusion criteria of the participants, which should have been submitted recently to different treatment modalities for drooling (within a maximum period of 6 months). Thus, the primary impact of the treatments is related to the development of white spot lesions and not caries lesions with cavitation, which tends to progress slowly.

As previously described, individuals with CP present lower SFR associated with higher SO (27) values, which are potential risk factors for dental caries (4) and gingivitis (28). In this context, the surgical procedure was the most harmful treatment modality observed in this study, resulting in a higher prevalence of WSL compared to the other treatments. The homogeneity among the groups, regarding oral hygiene (as measured by the OHI-S), allows us to state that the difference in WSL observed in G3 is due to the reduction of SFR and increase of SO.

Although there was no pairing in relation to the number of children and adolescents submitted to different treatment modalities in relation to gender and age, the adjusted multivariate regression analysis showed no statistical difference in relation to the presence of active WSL among children and adolescents with CP.

One significant problem experienced by participants who underwent treatment for drooling was the appearance of active WSL. This condition requires immediate non-invasive treatment by application of fluoride varnish or occlusal sealants with fluoride-releasing material, such as the latest generation of glass-ionomer cements. If patients’ initial lesions are not controlled, they may progress toward cavitation, painful sensation, difficulty in chewing, and the need for functional aesthetic rehabilitation.

According to the data of the last Brazilian Oral Health Survey (SB Brazil 2010), healthy children living in São Paulo, Brazil, present dental caries experience of 1.99 at the age of 5 years and 1.41 at the age of 12 years (29). Considering that the mean age presented by the participants of the present investigation was approximately 10 years old, and individuals with CP present a significant delay in tooth eruption, it can be inferred that the dental caries experience was similar in CP and in healthy children and adolescents.

Limitations of this study include the design (cross-sectional), the convenience sample, and the location: a reference rehabilitation center. Nevertheless, regarding the treatment modalities discussed in this study, surgical treatment demonstrated to be responsible for the caries risk of participants, due to the reduced SFR, increased SO values, and number of WSL. In this context, it is essential to include dental surgeons in multi-disciplinary medical teams (18,30), in order to prevent and treat the early signs of damage caused by treatments for drooling and to maintain individuals’ follow-ups to promote oral health. Moreover, caregivers of CP individuals should be informed about the oral health consequences of drooling treatment, such as difficulty in chewing and swallowing, thick saliva, and halitosis.

## Conclusions

Children and adolescents with cerebral palsy who have undergone surgical treatment for drooling exhibited higher number of white spot lesions compared to the other treatments, due to lower SFR rate, higher SO values and presence of WSL.

## References

[1] Rosenbaum P, Paneth N, Leviton A, Goldstein M, Bax M, Damiano D (2007). A report: the definition and classification of cerebral palsy April 2006. Dev Med Child Neurol Suppl.

[2] Erasmus CE, van Hulst K, Rotteveel JJ, Willemsen MA, Jongerius PH (2012). Clinical practice: swallowing problems in cerebral palsy. Eur J Pediatr.

[3] Dawes C (2008). Salivary flow patterns and the health of hard and soft oral tissues. J Am Dent Assoc.

[4] Santos MT, Ferreira MC, Mendes FM, de Oliveira Guaré R (2014). Assessing salivar osmolality as a caries risk indicator in cerebral palsy children. Int J Paediatr Dent.

[5] Reid SM, McCutcheon J, Reddihough DS, Johnson H (2012). Prevalence and predictors of drooling in 7- to 14-year-old children with cerebral palsy: a population study. Dev Med Child Neurol.

[6] Blasco PA, Allaire JH (1992). Drooling in the developmentally disabled: management practices and recommendations. Consortium on Drooling. Dev Med Child Neurol.

[7] Hegde AM, Pani SC (2009). Drooling of saliva in children with cerebral palsy-etiology, prevalence, and relationship to salivary flow rate in an Indian population. Spec Care Dentist.

[8] Tahmassebi JF, Curzon ME (2003). The cause of drooling in children with cerebral palsy -- hypersalivation or swallowing defect?. Int J Paediatr Dent.

[9] Santos MT, Ferreira MC, Leite MF, Guaré RO (2011). Salivary parameters in Brazilian individuals with cerebral palsy who drool. Child Care Health Dev.

[10] Nunn JH (2000). Drooling: review of the literature and proposals for management. J Oral Rehab.

[11] Yam WK, Yang HL, Abdullah V, Chan CY (2006). Management of drooling for children with neurological problems in Hong Kong. Brain Dev.

[12] Dias BL, Fernandes AR, Filho HS (2016). Sialorrhea in children with cerebral palsy. J Pediatr.

[13] Miranda-Rius J, Brunet-Llobet L, Lahor-Soler E, Farré M (2015). Salivary Secretory Disorders, Inducing Drugs, and Clinical Management. Int J Med Sci.

[14] Reddihough D, Erasmus CE, Johnson H, McKellar GM, Jongerius PH, Cereral Palsy Institute (2010). Botulinum toxin assessment, intervention and aftercare for paediatric and adult drooling: international consensus statement. Eur J Neurol.

[15] Noonan K, Prunty S, Ha JF, Vijayasekaran S (2014). Surgical management of chronic salivary aspiration. Int J Pediatr Otorhinolaryngol.

[16] Hallett KB, Lucas JO, Johnston T, Reddihough DS, Hall RK (1995). Dental health of children with cerebral palsy following sialodochoplasty. Spec Care Dentist.

[17] Ferraz Dos Santos B, Dabbagh B, Daniel SJ, Schwartz S (2016). Association of onabotulinum toxin A treatment with salivary pH and dental caries of neurologically impaired children with sialorrhea. Int J Paediatr Dent.

[18] Little SA, Kubba H, Hussain SS (2009). An evidence-based approach to the child who drools saliva. Clin Otolaryngol.

[19] Greene JC, Vermillion JR (1964). The Simplified Oral Hygiene Index. J Am Dent Assoc.

[20] (2013). Oral health surveys: basic methods. 5th ed..

[21] (2005). Criteria Manual: International Caries Detection and Assessment System (ICDAS II).

[22] Christensen D, Van Naarden Braun K, Doernberg NS, Maenner MJ, Arneson CL, Durkin MS (2014). Prevalence of cerebral palsy, co-occurring autism spectrum disorders, and motor functioning - Autism and Developmental Disabilities Monitoring Network, USA, 2008. Dev Med Child Neurol.

[23] Kok SE, van der Burg JJ, van Hulst K, Erasmus CE, van den Hoogen FJ (2016). The impact of submandibular duct relocation on drooling and the well-being of children with neurodevelopmental disabilities. Int J Pediatr Otorhinolaryngol.

[24] Kok SE, Erasmus CE, Scheffer ART, van Hulst K, Rovers MM, van den Hoogen FJA (2018). Effectiveness of submandibular duct relocation in 91 children with excessive drooling: A prospective cohort study. Clin Otolaryngol.

[25] Melgar R A, Pereira J T, Luz P B, Hugo F N, Araujo F B (2016). Differential impacts of caries classification in children and adults: a comparison of ICDAS and DMF-T. Braz Dent J.

[26] Pitts N (2004). "ICDAS" – an international system for caries detection and assessment being developed to facilitate caries epidemiology, research and appropriate clinical management. Community Dent Oral Epidemiol.

[27] Ruiz LA, Diniz MB, Loyola-Rodriguez JP, Habibe CH, Garrubbo CC, Santos MTBR (2018;1). A controlled study comparing salivary osmolality, caries experience and caries risk in patients with cerebral palsy. Med Oral Patol Oral Cir Bucal.

[28] Santos MT, Ferreira MC, Guaré RO, Diniz MB, Rösing CK, Rodrigues JA (2016). Gingivitis and salivary osmolality in children with cerebral palsy. Int J Paediatr Dent.

[29] (2010). National Survey of Oral Health 2010 (SB 2010): survey on the oral health conditions of the Brazilian population 2010. Main results. Ministry of Health.

[30] Dougherty NJ (2009). A review of cerebral palsy for the oral health professional. Dent Clin North Am.

